# Distribution of Breeding Population and Predicting Future Habitat under Climate Change of Black-Necked Crane (*Grus nigricollis* Przevalski, 1876) in Shaluli Mountains

**DOI:** 10.3390/ani12192594

**Published:** 2022-09-28

**Authors:** Mingming Li, Huaming Zhou, Jun Bai, Taxing Zhang, Yuxin Liu, Jianghong Ran

**Affiliations:** 1 Key Laboratory of Bio-Resources and Eco-Environment of Ministry of Education, College of Life Science, Sichuan University, Chengdu 610064, China; 2 Ganzi Tibetan Autonomous Prefecture Forestry Science Institute, Kangding 626000, China; 3 Department of Science and Technology Consulting Service, Forestry Exploration and Design Institute of Sichuan, Chengdu 610084, China

**Keywords:** black-necked crane (*Grus nigricollis*), climate change, flagship species, Shaluli Mountains, distribution change

## Abstract

**Simple Summary:**

The black-necked crane is the only crane species that breeds on the Qinghai–Tibetan plateau, and habitat destruction and loss are the major threats to it. The Shaluli Mountains, located in a biological hotspot, is an important breeding site for the central population of the black-necked crane, but its population size and distribution, as well as shifts in distribution under climatic change, remain unknown. Therefore, we conducted a field survey and modeled the breeding black-necked crane habitat with a species distribution model in the region. The results showed that the number of breeding black-necked cranes in the area was about 200, and they were mainly distributed in a gentle meadow and wetland along the lake. Climate change will increase the potentially suitable habitat area, with a trend of northwestward expansion and upward migration, as well as an increase in habitat suitability and connectivity. In addition, more conservation gaps will arise, but the conservation role of existing protected areas will not decline. Therefore, we suggest strengthening the long-term monitoring of black-necked cranes and wetland resources in this area.

**Abstract:**

Climate change is affecting biodiversity by altering the geographical distribution range of species, and this effect is amplified in climate-sensitive areas. Studying the geographic distribution of flagship species in response to climate change is important for the long-term conservation of species and the maintenance of regional biodiversity. Therefore, we collected field survey records from 2016 to 2020 and conducted field surveys of black-necked cranes in the Shaluli Mountains (SLLMs) in May–June and August–October 2021; 103 breeding records were acquired totally, and the geographical distribution range under the current and four future climate scenarios was modeled with the MaxEnt model to predict the impact of climate change on its distribution and habitat quality. The results showed that 152 black-necked cranes were surveyed in seven counties of SLLMs in total; the estimated number of black-necked cranes in the entire SLLMs was about 200. The currently suitable habitat area is 27,122 km^2^, mainly distributed in gentle meadows and wetland habitats along the lake where the Annual Mean Temperature is −1 °C and the Mean Diurnal Range (16 °C) and Precipitation Seasonality (105) are comparatively large. Furthermore, the breeding range would expand to varying degrees under future climate scenarios and showed a migration trend toward the northwest and higher elevation. Besides, as time goes by, the habitat for black-necked cranes in SLLMs would become more homogeneous and more suitable. The conservation effectiveness of the existing reserve network would keep stable with climate change, although there are large conservation gaps between protected areas, and these gaps will gradually expand over time. Overall, this study provides a preliminary understanding of the population and distribution and predicts the future distribution of black-necked cranes in the SLLMs. It also demonstrates the importance of SLLMs for protecting the central population of black-necked cranes and maintaining regional biodiversity. Therefore, we recommend long-term monitoring and conservation of the black-necked crane population and wetland resources in the region.

## 1. Introduction

Since 1900, 477 vertebrates including 69 mammal species, 80 bird species, 24 reptiles, 146 amphibians, and 158 fish have become extinct according to conservative estimations [1]. Climate change is one of the greatest threats to global biodiversity [2], in coming decades it is projected to be more serious, in turn, loss of biodiversity can adversely affect the climate, for example, deforestation increases the atmospheric abundance of carbon dioxide, and thus contributes to the greenhouse effect [3]. The change in the geographical distribution of species is a notable consequence of climate change [4]; one-sixth of extant species are estimated to be threatened with extinction due to climate-related range reduction [5,6]. We cannot protect every species meticulously due to limits of budget and staff [7], while the protection of flagship or umbrella species can reduce conservation costs and improve conservation effectiveness, thus it is often used as a conservation strategy in regional biodiversity conservation [8,9,10]. Flagship species are those that when conserved in situ result in the conservation of a significant number of other species across a wide array of taxonomic groups and in functioning natural systems [11] which are important for maintaining regional biodiversity [12,13]. The giant panda (*Ailuropoda melanoleuca* David, 1869) is a flagship species for biodiversity conservation; numerous rare plants and animals distributed in the same area as the giant panda have been protected in recent years [14,15,16]. In addition, choosing a flagship species strategically for a conservation marketing campaign will aid the protection of the species’ entire ecosystem [17]. For example, in Brazil, compared to communicating to the public to improve the conservation of threatened rainforest habitats, the interest, and pride in a spectacular national animal, such as the Golden Lion Tamarin (*Leontopithecus rosalia* Linnaeus, 1766) is easier to generate [18]; in Queensland, Australia, public concern for riverine habitats increased by 5.51 times with exposure to an education and outreach campaign featuring the platypus (*Ornithorhynchus anatinus* George Shaw, 1799) [19]. Meanwhile, in Japan, respondents showed a higher willingness to pay for the conservation of the red-crowned crane (*Grus japonensis* Müller, 1776) than the ecosystem services provided by its wetland habitat [20]. Studying the effects of climate change on the distribution of flagship species is vital to understanding the potential conservation issues and developing coping strategies, as well as maintaining regional biodiversity [21].

The black-necked crane (*Grus nigricollis* Przevalski, 1876) is the only one of fifteen crane species in the world that lives its entire life on the plateau [22,23] and is currently listed as “Near Threatened”, according to the IUCN Red List of Threatened Species [24]. Black-necked cranes breed in the Qinghai–Tibet Plateau and Ladakh and overwinter in low-altitude areas of the Qinghai–Tibet Plateau, Yunnan–Guizhou Plateau, Bhutan, and southern Tibet in China [25]. They play a crucial role in maintaining the biodiversity of the plateau wetland ecosystem as a flagship species and environmental indicator in Qinghai–Tibet Plateau alpine wetland ecosystems [26,27]. The black-necked crane tends to select different types of habitats in different districts. For instance, breeding black-necked cranes are concentrated in the plateau meadows, swampy meadows and marshes of Tibet, Xinjiang, eastern Qinghai and northern Sichuan, where herbaceous plants are abundant [23,28]. Black-necked cranes prefer to nest in mounds (or small islands) in shallow-water wetlands with low vegetation and use the deep mud layers near their nest to escape from predators or to prevent the nest from being flooded [29,30]. In overwintering areas, they prefer the marshes, lakeshore meadows, shoals, open warm rivers, and leeward wetlands [31,32,33,34]. These habitats are closely related to wetlands, these being the perfect sites for food, energy, and shelter for breeding colonies of black-necked cranes. Maintaining existing wetland areas as well as creating additional wetlands is key to their survival and potential expansion. Against the backdrop of global climate change, the Tibetan Plateau has undergone extreme changes [35]. Rising temperatures have led to the melting of numerous glaciers and permafrost, increased river runoff and rising lake levels [36], while precipitation has also increased year by year and has shown a gradual decrease from southeast to northwest [37]. The warm and humid hydrothermal conditions have been creating mass wetlands. However, previous studies have ignored the importance of particular habitats (plateau wetland ecosystem) to the continued existence of black-necked cranes [38], and also lacked research on habitat range shift and the associated impact mechanisms under the climatic change. Therefore, understanding the distribution range and changes in the black-necked crane habitat under a climate background can provide support for the conservation of the black-necked crane and local biodiversity [27].

Black-necked cranes are usually divided into Eastern, Western, and Central geographical subpopulations for the different wintering grounds [39]. The population size, habitat selection, migration, and overwintering of the Eastern population had already been researched clearly in previous studies [28,40,41,42,43,44], while the central population which is the smallest population of the three geographical subpopulations, has been studied scantily because of dispersed breeding sites, inconvenient transportation and harsh climate [45,46,47]. More seriously, surveys and studies of the central populations in the Shaluli Mountains (SLLMs) were the weakest, with only sites of distribution and numbers of localities being reported sporadically at present [22,30]; the knowledge of distribution, habitat, and population size of the entire breeding population was still lacking [48]. The SLLMs, in which the central breeding population is located, are the major component of the Southwest Mountains, one of the world’s 36 biodiversity hotspots [22,49], two of nine global migratory routes for migratory birds (Central Asia Flyway and East Asia-Australasia Flyway) pass through the region (https://www.eaaflyway.net/the-flyway/ (accessed on 9 June 2022)), which is of high biodiversity conservation value and also sensitive to climate change [35,50]. In this region, the swampy wetlands are mainly located in and around Haizishan in the central part of SLLMs, and less in the northern and southern areas, in addition, there are plentiful lake wetlands in Haizishan [51]. These wetlands are critical breeding areas for black-necked cranes. As the climate warms, rainfall increases, glacial melting, etc. [36,37,52], the SLLMs will become wetter, and more meadow wetlands will appear in the north. Therefore, understanding the current distribution of the breeding black-necked cranes in SLLMs and predicting the changes in the range under future climate scenarios can better protect the central population of black-necked cranes and provide a scientific basis for formulating corresponding conservation strategies in response to climate change, contributing to the conservation of regional and global biodiversity.

In this study, the numbers and distribution of field populations of black-necked cranes in SLLMs were surveyed in May–June and August–October 2021; the species distribution model for breeding black-necked cranes in SLLMs was constructed with the MaxEnt model based on the breeding locations obtained from field surveys, climatic data from Coupled Model Intercomparison Project Phase 6 (CMIP6) and other environmental variables. The aims of the present study were (1) to understand the population numbers of breeding black-necked cranes in SLLMs preliminarily; (2) to predict the geographical range of black-necked cranes in SLLMs under the current climate scenario and the impact factors; (3) to predict changes of distribution range and landscape features under future climate change; (4) to explore existing conservation gaps and future changes.

## 2. Materials and Methods

### 2.1. Study Area and Field Survey

**Figure 1:**
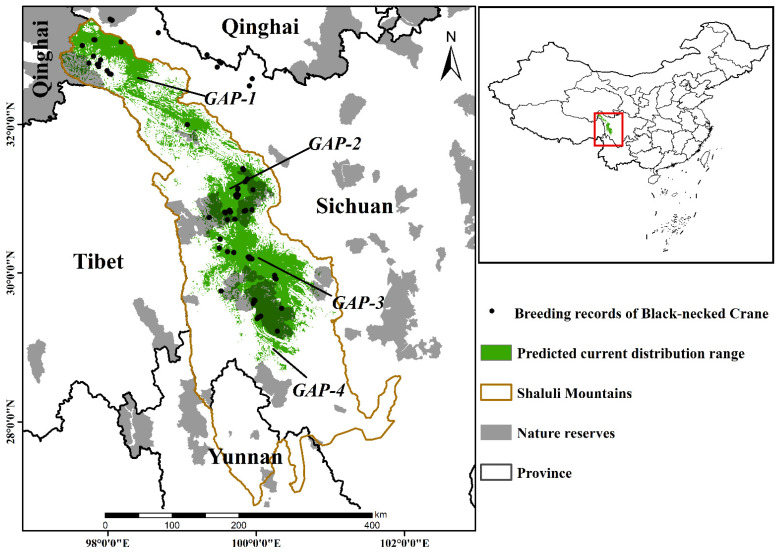
The predicted current distribution range of the black-necked crane and the conservation gaps (GAP-1,2,3,4) under the current nature reserves network in the Shaluli Mountains.

The Shaluli mountains are one of the major parts of the Hengduan Mountains one of the 36 biodiversity hotspots around the world [49,53], located in the west of Sichuan, the southeast part of the Qinghai–Tibet Plateau (26.88° N–33.42° N, 97.35° E–101.94° E) (Figure 1). The topography is complex with distinct vertical zones, from dry thermal valleys to alpine permafrost, while the climates vary dramatically, with an average annual temperature difference of up to 20.2 °C [49]. The same as other mountains in the Heng duan Mountains [54,55,56], the complex and varied natural conditions of SLLMs had given rise to a rich variety of vegetation types and animal populations [49,57]. In addition, it is an important breeding area and migration stopover for the central population of black-necked cranes [22,30].

The recordings of historical surveys (2016–2020), published literature [22,48], the China Bird Report Center (https://www.birdreport.cn/ (accessed on 25 November 2021)) (2014–2021), and the Global Biodiversity Information Facility (GBIF, https://www.gbif.org/ (accessed on 9 November 2021)) (2001–2020) show that there were breeding records of black-necked cranes in seven counties of SLLMs. Moreover, as indicated by many studies, the breeding habitats black-necked cranes preferred are mainly in remote mountainous areas’ highland lakes, river and marsh wetlands, and surrounding meadows [38,43,58]. Therefore, some areas of potential black-necked crane distribution were delineated in advance on electronic maps based on historical point locations and vegetation types. Moreover, we conducted a survey of breeding black-necked cranes in these areas from May to June 2021 and August to October 2021 with a combination of direct counting and visiting survey methods. (1) Direct counting method: Black-necked cranes are large and easy to identify in the field, so monoculars and binoculars were used as tools to conduct direct observation counts of black-necked cranes in the survey area by vehicle and on foot. Once the target specie was found, its latitude and longitude, number, population composition, habitat types, etc., were recorded. (2) Visiting survey method: Interviews were conducted with staff of the forestry system, villagers, and monks at the survey areas by showing pictures of black-necked cranes, with one person as an independent interviewee. Information on place and time of occurrence, numbers, population composition, nest sites, and information on human activities, was verified by the staff and marked on the satellite maps. The home range of the black-necked crane during breeding was 143.38 ± 34.46 ha [59], thus, the maximum number of black-necked cranes counted in a single survey plus the number of newborn cranes survey that year was regarded as the number of black-necked cranes in an area without considering the spread of black-necked cranes.

### 2.2. Habitat Predicting

#### 2.2.1. Species Occurrence Records

We gathered 103 breeding records of the black-necked crane in SLLMs and surrounding areas from 2016 to 2021 from field surveys (Table S1). For the strong mobility of black-necked cranes, we made a 100 km buffer outward from the boundary of SLLMs and used all distribution points falling into this area for model construction. To reduce the possibility of model overfitting, we used spThin which was an R package to prescreen the retained records and kept a minimum nearest neighbor distance greater than or equal to 1 km [60] between any two distribution points, which approximates the minimum home range of the black-necked crane during the breeding period [59]. Finally, 71 presence points of the black-necked crane remained and were used to build an ecological niche model.

#### 2.2.2. Environmental Variables

To capture the current breeding habitat in our species distribution models (SDMs), we collected (a) 19 bioclimatic variables; (b) surface elevation data from WorldClim (http://www.worldclim.org/ (accessed on 15 March 2022), v 2.1, 1970–2000); (c) global land cover data from the Climate Data Store (CDS, https://cds.climate.copernicus.eu/ (accessed on 15 March 2022)), which are divided into 22 classes. We combined several of them to obtain a total of 12 categories (farmland, agroforestry, broad-leaved forest, coniferous forest, shrub, meadow, desert, wetland, urban areas, bare areas, water bodies and, glacier); (d) the monthly mean net primary productivity from April to November in 2016 from NASA Earth Observation (https://neo.gsfc.nasa.gov/ (accessed on 16 March 2022)); (e) two layers of slope and aspect were extracted from the surface elevation data in (b). To explore the preference of black-necked cranes for shady and sunny slopes, we reclassified the slope direction layer into five categories: 1. Flat slope (−1), 2. Shady slope (0–45 and 315–360), 3. Half shaded slope (45–135), 4. Sunny slope (135–225), 5. Half sunny slope (225–315); (f) four layers of distance to the nearest perennial rivers, lakes, residential locations (villages and rural settlements), and roads were produced using the Euclidean Distance Tool of ArcGIS. The basic vector layers of rivers, lakes, roads and residential locations were downloaded from OpenStreetMap (https://www.openstreetmap.org/ (accessed on 7 April 2022)). See Table S2. for a complete overview and detailed information on all variables.

For future climate simulations, we selected the same set of climate variables for four time periods (2021–2040, 2041–2060, 2061–2080, 2081–2100) of four Shared Socioeconomic Pathways (SSPs) (SSP1-2.6, SSP2-4.5, SSP3-7.0, and SSP5-8.5). Contrasting with Representative Concentration Pathways (RCPs), the SSPs were intended to describe worlds in which societal trends result in making the mitigation of, or adaptation to, climate change harder or easier, without explicitly considering climate change itself [61]. The medium-resolution National (Beijing) Climate Center Climate System Model (BCC-CSM2-MR) was chosen as the global climate model (GCM), which can perform better in many aspects including the tropospheric air temperature and circulation at global and regional scales in East Asia and climate variability at different timescales [62].

Each environmental variable resampled the grid size to 1 km ∗ 1 km, using a bilinear interpolation method for the continuous variables and the nearest neighbor assignment for the categorical vegetation layer [63].

#### 2.2.3. Model Procedure

The Maximum Entropy Model was usually employed to simulate the habitat suitability distribution due to the advantages of using presence-only data and performing well with incomplete data, small sample sizes, and gaps [64]; because of the high accuracy and migration ability of the model, it was widely used to predict habitat change of species under different climatic backgrounds as well. Therefore, we used MaxEnt 3.4.1 [65] to construct a distribution model of the black-necked crane in this study. The Band Collection Statistics Tool in ArcGIS 10.2 was used to remove highly correlated variables (|r| > 0.8) from 19 bioclimatic variables for the model fitting process and interpretation of results to eliminate the influence of collinearity between variables on the model fitting process and interpretation of results (Table S3) [66]. Eventually, 16 predictors were preserved to build our SDMs, including five geographical variables (altitude, aspect, slope, and distance to rivers/lakes), seven current bioclimate variables (Bio_1/2/7/8/12/15/17), two anthropogenic variables (distance to roads/settlements), and two vegetation factors (land cover and NPP). 

The regularization multiplier and the combination of features may influence the predictive performance and accuracy of the model [67,68], so we optimized the Regularization Multiplier and Feature Combination by using the Wallace v1.0.6 package in R [69]. We evaluated five feature combinations: L (linear), LQ (linear, quadratic), H (hinge), LQH (linear, quadratic, hinge), and LQHP (linear, quadratic, hinge, product), while regularization multipliers were sat as 0.5 to 4 with a step value of 0.5. Finally, there were 40 models generated by the above procedure, and the models with the lowest AIC (Akaike Information Criterion) were identified as optimal among the candidate models [70,71].

The model whose feature combination and regularization multiplier were LQ + 1 had the lowest AIC (Table S4). Thus, we used this feature combination and regularization multiplier to construct a MaxEnt model for the current black-necked crane range. All the remaining distribution points of the black-necked crane were imported into the MaxEnt software, and the running parameters were set to randomly select 25% of the distribution points as the test data and 75% of the distribution points as the training data for a model accuracy analysis set, the output format was “Cloglog” [72], the output file type was “ASCⅡ”, the maximum iteration mode was set “Bootstrap” and the number of repetitions was 20. To determine the importance of each environmental variable, the jackknife test was performed [73]. Three threshold-independent methods (AUC, Maximum Kappa and TSS) [74,75,76,77] were selected to evaluate the model; the output values of the model were continuous [78]. The AUC was indicated with the mean values of training and test AUC of 20 model replicates. Moreover, the maximum Kappa and TSS were calculated with the “Presence Absence” package in R 4.4.1 [79].

For projecting future distributions, we added a set of variables excepting the climate variables were from the future; the others were kept consistent with the variables of the current model. Such predictor combinations consisting of dynamic climate variables and other static variables could help enhance the performance of our SDM under future climate scenarios [80].

### 2.3. Spatial Analysis 

The maximum test sensitivity plus specificity was regarded as the threshold to produce binary presence/absence distribution maps. This method is proven to not be affected by the ratios of the number of known presences to the random background samples [81,82]. In this study, the threshold corresponding to the habitat suitability index (HSI) was 0.17. Therefore, we produced continuous prediction maps for the predicted presence area (HIS ≥ 0.17) under current and future climate scenarios. Then, we calculated the mean elevation, and mean HSI for these models to study the changes in habitat suitability and distributing altitude of the black-necked crane in SLLMs under future climate scenarios. Additionally, the patch density [83] and clumping index [84] were calculated by FRAGSTATS 4.2 [85] to assess the degree of habitat fragmentation. They were suitable for comparisons of fragmentation among different climate scenarios [6]. Finally, we overlapped current and future suitable habitats with the borders of established nature reserves to explore the conservation effectiveness and gaps in these reserves for protecting the black-necked crane under future climate change scenarios using ArcGIS 10.2.

## 3. Results

### 3.1. Current Breeding Numbers and Habitat Ranges

A total of 152 black-necked cranes were recorded in the two field surveys, of which 141 were adults and 11 were juveniles (Table 1). The largest number of breeding black-necked cranes were found in Daocheng and Litang, accounting for 50.66% of the total number of observations, followed by Shiqu, accounting for 25.00%, then Baiyu and Xinlong accounting for 19.74% of the total number of surveys, while Batang and Dege had the lowest numbers, with four and three individuals, respectively, accounting for only 4.6% of the total number of surveys. Besides, 46–53 individuals were recorded by visiting survey (Table 1). Consequently, with the addition of the number of the visiting survey, we estimated preliminary that the breeding population of black-necked Cranes in the entire SLLMs was approximately 200 individuals.

**Figure 2:**
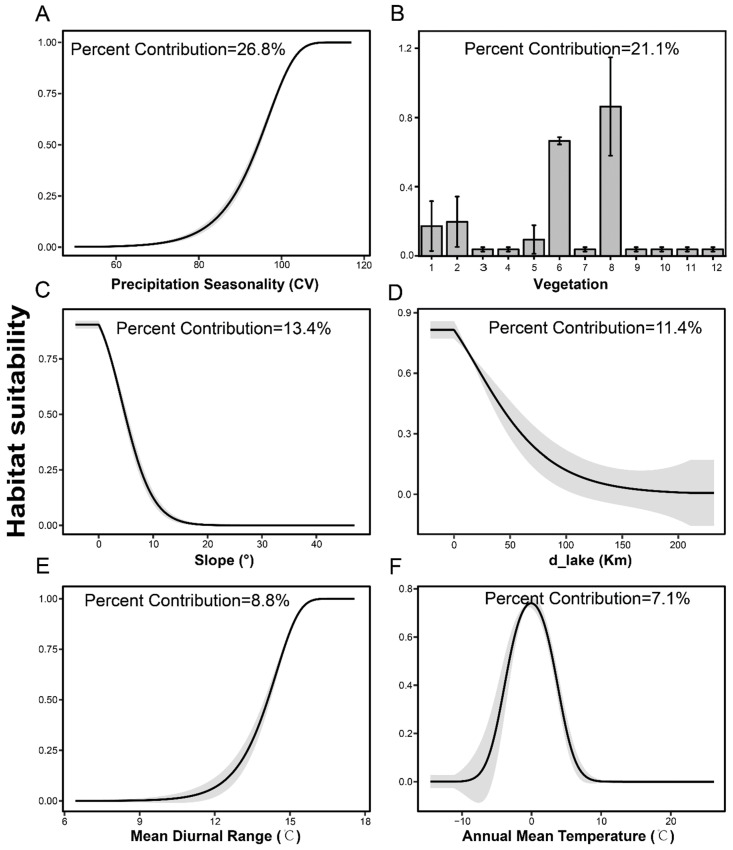
Response curves of habitat suitability for the black-necked crane (vertical axis) to Precipitation Seasonality(**A**), Vegetation (**B**), Slope (**C**), d-lake (**D**), Mean Diurnal Range (**E**), and Annual Mean Temperature (**F**) showing as means (black lines) with standard deviation (SD, gray buffers) except Vegetation (**B**). The abscissa axis’s 1–12 of Vegetation (**B**) refers to (farmland, agroforestry, broad-leaved forest, coniferous forest, shrub, meadow, desert, wetland, urban areas, bare areas, water bodies, and glacier) respectively.

The average value of training AUC and test AUC, maximum Kappa, and maximum TSS for the model built in the Section 2.2.3 were 0.953, 0.939, 0.634, and 0.777, respectively. These evaluation indexes indicated that the MaxEnt model for the current distribution range of the black-necked crane performed well [86,87,88]. The contribution of each environmental variable to the model showed Precipitation Seasonality (bio15) contributed (26.8%) most to the model, followed by vegetation (21.10%), slope (13.4%), distance to lakes (d-lake) (16.33%), Mean Diurnal Range (bio2) (8.8%) and Annual Mean Temperature (bio1) (9.70%). These six predictors contributed 88.6% to the model (Table S5). Response curves showed the quantitative relationship between the important environmental predictors and HSI for the black-necked crane. In terms of habitat, black-necked cranes prefer wetland and meadow habitats with gentle slopes along lakes (Figure 2B–D), and for hydrothermal conditions, they prefer the area with a low Annual Mean Temperature (−1 °C) (Figure 2F), relatively large Mean Diurnal Range (16 °C) (Figure 2E) and comparatively large Precipitation Seasonality (105) (Figure 2A).

According to model predictions, the suitable area for black-necked cranes in SLLMs under current climate conditions was 27,122 km^2^, accounting for 26.32% of the entire SLLMs (Table S6). These suitable habitats were mainly concentrated in the Haizishan National Nature Reserve, the Sichuan Chaqinsongduo White-lipped deer National Nature Reserve and the Xionglongxi Wetland Nature Reserve which were in the central part of SLLMs (Figure 1).

### 3.2. Predicting Suitable Habitats in Future

**Figure 3:**
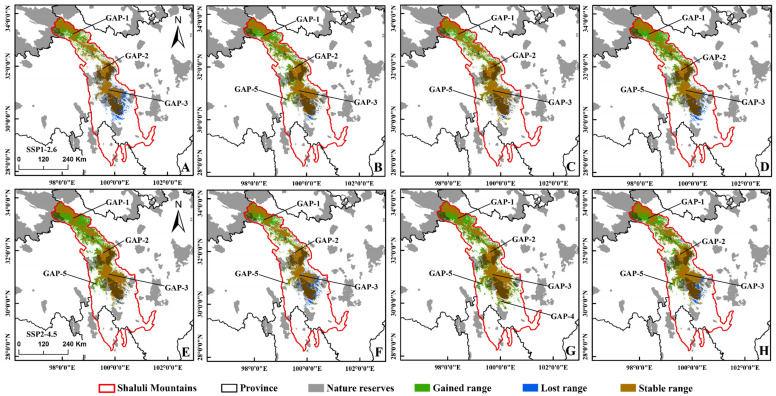
The potential distribution of suitable areas for the black-necked crane under the climatic conditions of shared socio-economic pathway 1–2.6 (top), 2–4.5 (bottom) during different periods of the 21st century: (**A**,**E**) 2021–2040, (**B**,**F**) 2041–2060, (**C**,**G**) 2061–2080, (**D**,**H**) 2081–2100.

**Figure 4:**
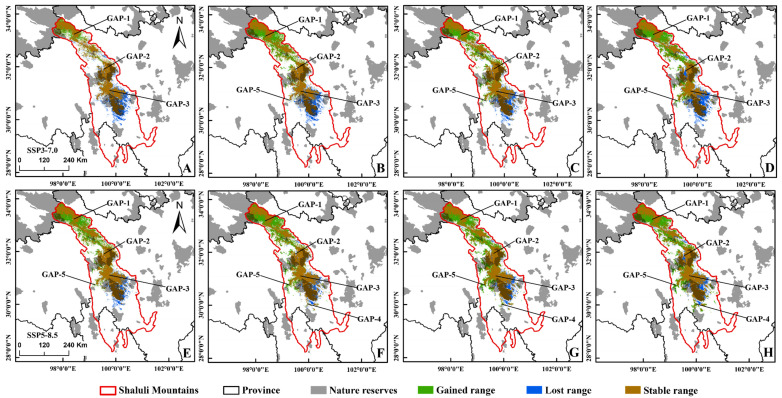
The potential distribution of suitable areas for the black-necked crane under the climatic conditions of shared socio-economic pathway 3–7.0 (top), 5–5.8 (bottom) during different periods of the 21st century: (**A**,**E**) 2021–2040, (**B**,**F**) 2041–2060, (**C**,**G**) 2061–2080, (**D**,**H**) 2081–2100.

**Figure 5:**
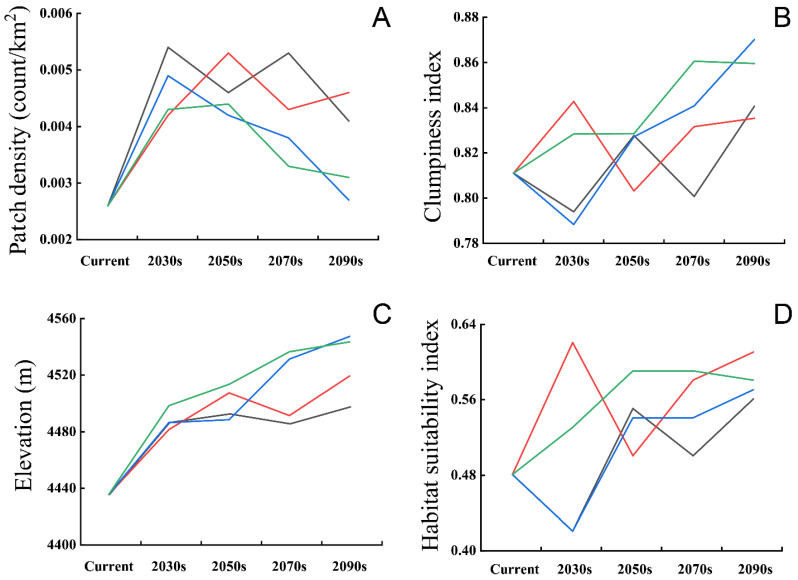
Effects of climate change on patch density (**A**), Clumpiness index (**B**), mean elevation (**C**), and mean habitat suitability index (**D**) of the black-necked crane distribution range, under shared socio-economic pathway SSP1-2.6 (black lines), SSP2-4.5 (red lines), SSP3-7.0 (blue lines) and SSP5-8.5 (green lines).

The models predicted a dramatic range shift in the horizontal and vertical distribution of the suitable habitat for black-necked cranes in SLLMs under four climate scenarios. In horizontal distribution, the suitable distribution would expand to the northwest, while the suitable habitat in the south would decrease, and more unsuitable habitats in the north would become suitable (Figure 3 and Figure 4). Especially in the SSP2-4.5, the suitable area for black-necked cranes was expected to increase by 47.56% compared with the 2070s (Table S6). In vertical distribution, the average altitude of suitable habitat for the black-necked crane increased under all climate scenarios, especially, would increase from the current 4435 m to 4547 m by 2081–2100 in the SSP3-7.0 (Figure 5C).

The suitability of the black-necked crane distribution range would improve with climate change, causing the average habitat suitability index of black-necked cranes in different scenarios to be significantly higher than in the current climate, at the end of this century (Figure 5D). The connectivity of black-necked cranes’ breeding habitat in SLLMs would be affected by climate change as well. Patch density of the distribution area showed a trend of increasing then decreasing under all climatic conditions (Figure 5A). The patch density for SSP3-7.0 and SSP5-8.5 decreased to be consistent with the current climate, and for SSP1-2.6 and SSP2-4.5 were slightly higher than the present climate, while the clumping index was higher than the present for all climate scenarios in the 2090s (Figure 5A,B). All the above suggested that the connectivity of future black-necked crane breeding habitats in SLLMs will vary with climate change but would generally become better under these climatic scenarios.

### 3.3. Conservation Gaps

**Figure 6:**
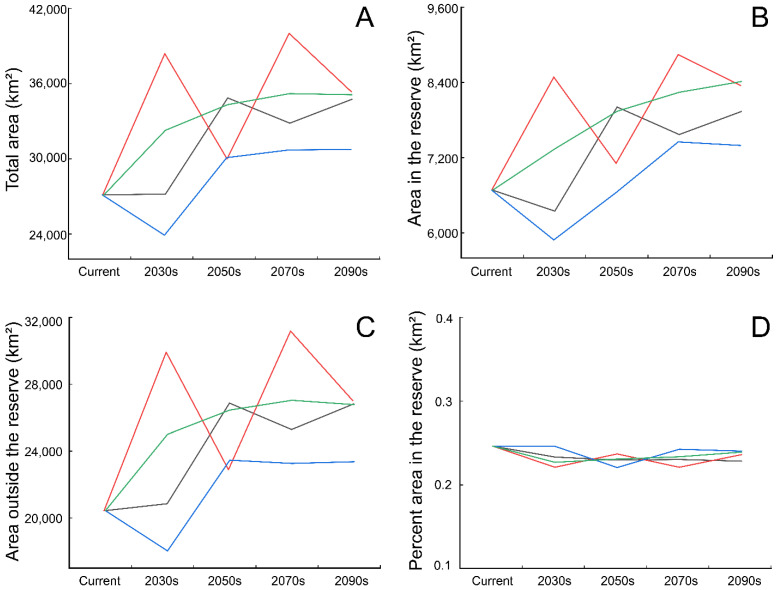
Effects of climate change on the total suitable habitat area (**A**), the suitable area within the protected area (**B**), the suitable area outside the protected area (**C**), and the proportion of protected area (**D**) for black-necked cranes in the Shaluli Mountains, under shared socio-economic pathway SSP1-2.6 (black lines), SSP2-4.5 (red lines), SSP3-7.0 (blue lines) and SSP5-8.5 (green lines).

Only 6683 km^2^, accounting for 24.64% of the suitable habitat for breeding black-necked cranes was protected by the existing conservation network in SLLMs at present, with most of them outside the nature reserves (Table S6). Over time, the suitable area within the reserve would be increasing (Figure 6B), while the area outside gradually increased too (Figure 6C). Existing conservation gaps were mainly located in the north (Figure 1 GAP-1), the middle (Figure 1 GAP-2/GAP-3), and the south (Figure 1 GAP-4) of SLLMs. As time goes on, GAP-4 will disappear, potentially suitable habitat areas at GAP-1 will increase greatly, and new conservation gaps (GAP-5) will emerge (Figure 4 and Figure 5).

## 4. Discussion

### 4.1. Current Breeding Numbers and Habitat Ranges

The breeding black-necked cranes were recorded in seven counties, which is consistent with the historical record [48]. However, the population was smaller and scattered. A total of 152 individuals were recorded, except for a group of 18 individuals, the rest of the records were mostly single breeding pairs, less than the number in Longbao National Nature Reserve, Yushu [45] or Zoige wetland, Sichuan [28,44]. The reason may be related to the area of the wetland. The total wetland area of Zoige is 5923.83 km^2^ [51], which is the largest among the three regions, so the number of black-necked cranes in Zoige is as high as 2600 [89]. The wetland area in SLLMs is much larger than that of the Longbao National Nature Reserve, which is 100 km^2^, but the number of black-necked cranes was still lower than that in the Longbao National Nature Reserve. The reason may there are large areas of scrub swamp wetlands in the SLLMs besides meadow swamps, and the habitat was more dispersed than that in the Longbao National Nature Reserve [45]. Scrub swamp wetlands may not afford larger populations by providing fewer food resources; 50.66% of individuals were surveyed in the Haizishan of Daocheng and Litang counties, this may be due to the abundant lakes and swampy wetlands in the Haizishan area with a wetland area of 1437 km^2^; in addition, the whole area was protected by the Haizishan Nature Reserve, which is remote and human activity is low [30]. Moreover, Haizishan was proved the major breeding site for black-necked cranes in SLLMs once more. We also found that the number of individuals in Shiqu is also higher, probably because Shiqu has a large area of wetlands, such as the Changsha Gongma National Wetland, which can provide enough food and places for black-necked cranes to live and breed [38]. In addition, black-necked cranes were likely to spread from Longbao, Yushu, which is only 50 km away to Shiqu, Sichuan [45].

The current distribution range of breeding black-necked cranes predicted by the model is mainly concentrated in the area north of the Haizishan (Figure S1), which is consistent with the field survey range. This further adds to our confidence that our model correctly predicts the current breeding range for the black-necked crane. When comparing the distribution range of black-necked cranes with that of other studies, we found that the distribution map on the IUCN did not include SLLMs, while the map from ICF included only the north part of SLLMs except for the Haizishan (Figure S1). IUCN is the most widely used endangered species evaluation system, in which the distribution range of species is an important basis for endangered species ranking [90], so the distribution range predicted in this study can provide a reference for the subsequent endangered species ranking and distribution range classification.

### 4.2. Projected Breeding Ranges under Climate Change Scenarios

The modeling results indicate that the suitable breeding ranges of the black-necked crane would gradually decrease in the south and increase in the north, with an overall approach to the interior of the Tibetan Plateau, assuming no adaptive change in habitat preferences, which is consistent with the findings of a 2018 study [82]. Climate change did not reduce the habitat area but increased gradually. The reason may be that with climate change, the northern SLLMs will become wetter due to glacial melt [52], and more wetlands will emerge to provide more food resources and habitat for black-necked cranes. In addition, continued warming would reduce the area of permanent glaciers in the mountains [91], and advance the phenological period of alpine vegetation [92,93], making the previously fragmented habitat continuous [94].

Previous studies demonstrated that the distribution of some specific species may exhibit poleward and upward range shifts that may occur with climate warming [95,96], here, our model showed the same result. The preference of black-necked cranes for alpine meadows is related to their species formation [97], and this preference will continue to influence their distribution. Meanwhile, the increase in distribution elevation due to climate change will expose black-necked cranes to lower anthropogenic disturbance, which may bring relevant prosperity to the black-necked crane population.

### 4.3. Conservation Implications and Outlook

Habitat suitability directly determines the population density of species, especially for the crane species that show strong territory requirements during the breeding period [98], while increased habitat suitability will provide more food resources per unit area and will be able to carry larger populations [29]. Model predictions indicate that habitat suitability in SLLMs will become higher in the future, so we speculate that the population of black-necked cranes in this region may gradually increase over time. The conservation gap analysis found that more potentially suitable habitats will occur in the conservation gap in the northern part of the SLLMs under future climate scenarios (Figure 3 and Figure 4, GAP-1), while those in the central part will not change much (Figure 3 and Figure 4, GAP-2/3) in the grazing core area with many traditional summer pastures. The reduction in snow and ice disasters may promote more livestock breeding in summer pastures [99], which could threaten black-necked cranes. Climate change causes migratory birds to advance their arrival and departure times in spring [100]. Therefore, climate change may lead to an earlier migration period for black-necked cranes, which has also been reported in other crane species [101]; the new suitable habitat may become a migratory stopover for many black-necked cranes and other waterfowl because studies have found that migratory birds follow changes in environmental resources [102]. The field survey found that breeding black-necked cranes prefer wetlands along the lake, this is consistent with our model results; a study in the Zoige, Sichuan showed the same conclusion [103], indicating that the distance from the lake is an important factor influencing the habitat selection of black-necked cranes. Black-necked cranes do not show a tendency to avoid livestock, but the activities of herdsmen and herding dogs, road construction, and drug collection have a greater impact on black-necked cranes during the breeding period, which may lead to a lower breeding success rate of cranes [104]. In addition, the increasing distribution elevation due to climate change will result in the loss of some suitable habitats at lower elevations, while black-necked cranes have a high nest site fidelity [105], so individuals currently breeding at lower elevations will face greater environmental stress.

To protect the black-necked crane better, especially for a newly discovered breeding site, we suggest the following for the conservation management of this local population. (1) Long-term monitoring of the population size in the area will be conducted, while the protection and monitoring of wetland resources will be strengthened, understanding of the dynamic changes to wetland resources and black-necked crane populations to adjust conservation strategies better. (2) The focus of conservation remains on protecting the suitable habitats in existing protected areas and suggested that conservation plots be established in the northern part of the SLLMs based on a township for the technical and political complexity in designing and establishing new nature reserves; technical training is also provided to local conservationists to protect and monitor the black-necked cranes once they appear. (3) Paying attention to the distribution of migrating black-necked cranes and setting up appropriate resupply points, and adjusting grazing areas to reduce disturbance following the actual situation during the spring and autumn migration seasons, in addition, planning the number of grazing areas reasonably, improving the standardization of the activities of people entering the mountains, and focusing on ecological restoration after major projects (e.g., Sichuan–Tibet Railway) throughout the distribution area of black-necked cranes, and strengthening the protection of existing suitable habitats at low altitude. (4) Monitoring of black-necked cranes’ activity patterns and reproduction behaviors should be conducted to improve our understanding of how black-necked crane reproduction will respond to climate change.

As the flagship species of the alpine wetland ecosystem, the black-necked crane should be the focus of our conservation efforts to maintain the wetland ecosystem and regional biodiversity and to actively investigate the biological interactions between the black-necked crane and waterbirds in the region, to preserve our migratory bird heritage and ultimately save the ecosystem to which we all belong. Furthermore, climate change increases the risk of extinction for species dependent upon a narrow range of habitats or that exhibit poor adaptation to climate [38,106]. The black-necked crane is a typical narrow-habitat-range species, its ability to adapt to climate change is not yet known, so it is important to study the climatic-niche evolution and divergent ecological selection mechanisms for understanding the distribution of suitable habitats under climate change more accurately [107]. In addition, a systematic study on the effects of climate change on the population size and habitat of the breeding and wintering areas at different spatial and temporal scales, understanding the ability to adapt to changes in climate and habitat structure, and identifying specific strategies adopted to cope with these changes, is crucial for maintaining the long-term survival of the black-necked crane in climate-sensitive regions [38].

## 5. Conclusions

We surveyed the number and distribution of black-necked cranes during breeding in the SLLMs. The results show that the number of breeding black-necked cranes in the SLLMs is about 200, and they are mainly distributed in the central part of the SLLMs; with climate change, the area of suitable habitat in the Shaluli Mountain region will increase, and the altitude of the distribution of black-necked cranes will rise. In addition, the suitability of the whole habitat will increase, thus the number of black-necked cranes in the region is expected to increase. Climate change will not reduce the conservation role of the current protected areas, and the new conservation openings are mainly in the northern region of the SLLMs. Potential habitat expansion due to climate change will create additional management and conservation challenges as well as increased conservation and management investments; therefore we recommend long-term monitoring and protection of the black-necked crane and wetland resources in the area, which is important for maintaining the central population of black-necked cranes as well as regional biodiversity.

## Figures and Tables

**Table 1:** The numbers and distribution of black-necked cranes in the Shaluli mountains.

Distribution Area	Field Survey		Percentage (%)	Visiting Survey
	Adults	Subadult		
Daocheng county	36	0	23.69	23–29
Litang county	37	4	26.97	17–18
Batang county	4	0	2.63	
Baiyu county	14	1	9.87	
Xinlong county	14	1	9.87	2
Dege county	2	1	1.97	
Shiqu county	34	4	25.00	4
Total	141	11		46–53
	152			

## Data Availability

Not applicable.

## Supplementary Materials

The following supporting information can be downloaded at: https://www.mdpi.com/article/10.3390/ani12192594/s1 [24,108,109], Figure S1: Current distribution range for the black-necked crane using MaxEnt, compared with the distribution range maps from IUCN and ICF (International Crane Foundation); Figure S2: Distribution of breeding black-necked crane survey sites in the Shaluli Mountains; Table S1: Sources of Black-necked crane presence records; Table S2: Environmental variables were used in this study; Table S3: The correlation matrix of 7 final bioclimates for predicting the current distribution ranges of Black-necked cranes in Shaluli Mountains; Table S4: Selection of feature combination and regularization multiplier for optimizing MaxEnt model complexity; Table S5: Analysis of variable contributions for the current breeding habitats of the black-necked crane in Shaluli Mountains; Table S6: Area of predicted distribution range for the black-necked crane at present and under future climate scenarios.
