# Supplementary material for: Distribution of Breeding Population and Predicting Future Habitat under Climate Change of Black-Necked Crane (Grus nigricollis Przevalski, 1876) in Shaluli Mountains
Source: Animals (Basel). 2022 Sep 28;12(19):2594. doi: 10.3390/ani12192594 (PMC9558536; doi:10.3390/ani12192594)
Supplement: Supplementary file 1 [file animals-12-02594-s001.zip › animals-1871928-supplementary.pdf]

Table S1. Sources of Black-necked crane presence records.

Source	Survey region	Survey time	Survey Project	Presence records
Field surveys conducted by the authors	Ten counties in Sichuan Province	2021.5-6 2021.8-10	The special investigation on the breeding black-necked cranes in SLLMs	68
Field surveys conducted by the authors	Several counties in Sichuan and Qinghai Province	2021.7-8	The Second Tibetan Plateau Scientific Expedition and Research Program	12
Yang et al. (2020)1	Several counties in Sichuan	2016-2020	The usual survey	24

[1] Part of the data was reported in Lu et al. (2022).

References

1. Lu, B.G.; Luo, L.L.; Xie, F.; Ye, S.; Yang, N. Quality assessment of the breeding habitat of black-necked cranes in Baiyu County, Sichuan Province. Journal of Southwest Minzu University (Natural Science Edition). **2022**, *48*, 237-244.

Table S2. Environmental variables were used in this study. sixteen variables for predicting the current and future breeding range were bolded.

Environmental variables	Description	Data source
**WordClim_Bio1**	Annual Mean Temperature	http://www.worldclim.org/
**WordClim_Bio2**	Mean Diurnal Range (Mean of monthly (max temp - min temp))	
WordClim_Bio3	Isothermally (BIO2/BIO7) (×100)	
WordClim_Bio4	Temperature Seasonality (standard deviation ×100)	
WordClim_Bio5	Max Temperature of Warmest Month	
WordClim_Bio6	Min Temperature of Coldest Month	
**WordClim_Bio7**	Temperature Annual Range (BIO5-BIO6)	
**WordClim_Bio8**	Mean Temperature of Wettest Quarter	
WordClim_Bio9	Mean Temperature of Driest Quarter	
WordClim_Bio10	Mean Temperature of Warmest Quarter	
WordClim_Bio11	Mean Temperature of Coldest Quarter	
**WordClim_Bio12**	Annual Precipitation	
WordClim_Bio13	Precipitation of Wettest Month	
WordClim_Bio14	Precipitation of Driest Month	
**WordClim_Bio15**	Precipitation Seasonality (Coefficient of Variation)	
WordClim_Bio16	Precipitation of Wettest Quarter	
**WordClim_Bio17**	Precipitation of Driest Quarter	
WordClim_Bio18	Precipitation of Warmest Quarter	
WordClim_Bio19	Precipitation of Coldest Quarter	
**WorldClim_ELEV**	Elevation	
**Slope**	Slope (°)	
**Aspect**	Aspect (5 categories)	
**d-lake**	Euclidean distance from the lake	https://www.openstreetmap.org/
**d-river**	Euclidean distance from the river	
**d-road**	Euclidean distance from the road	
**d-settlement**	Euclidean distance from the river	
**NPP**	Net Primary Production	https://neo.gsfc.nasa.gov/
**Vegetation**	Vegetation type (12 categories)	https://cds.climate.copernicus.eu/

Table S3. The correlation matrix of 7 final bioclimates for predicting the current distribution ranges of Black-necked cranes in Shaluli Mountains.

**Bio1**	1						
**Bio2**	-0.55508	1					
**Bio7**	-0.78253	0.69395	1				
**Bio8**	0.79877	-0.37463	-0.40047	1			
**Bio12**	0.71103	-0.68672	-0.78211	0.4969	1		
**Bio15**	-0.01865	0.36303	0.10125	0.11112	-0.15772	1	
**Bio17**	0.38207	-0.48822	-0.50503	0.2267	0.6758	-0.50831	1
	**Bio1**	**Bio2**	**Bio7**	**Bio8**	**Bio12**	**Bio15**	**Bio17**

Table S4. Selection of feature combination and regularization multiplier for optimizing MaxEnt model complexity, the feature combination and regularization multiplier used in this study were bolded.

Feature combination	Regularization multiplier	AUC.train	AICc	Delta.AICc
L	0.5	0.903256338	1649.628775	25.58070235
L	1	0.899875352	1651.964167	27.91609352
L	1.5	0.896082394	1651.9648	27.91672651
L	2	0.894503521	1650.561347	26.51327443
L	2.5	0.894062676	1648.644009	24.59593611
L	3	0.893526056	1649.918098	25.87002545
L	3.5	0.892772535	1648.513033	24.46496031
L	4	0.891876761	1650.243747	26.19567439
LQ	0.5	0.925978169	1628.763243	4.715170315
**LQ**	**1**	**0.920250704**	**1624.048073**	**0**
LQ	1.5	0.917182394	1632.474773	8.426699936
LQ	2	0.914265493	1635.22723	11.17915686
LQ	2.5	0.909882394	1642.874203	18.82612993
LQ	3	0.906214789	1639.0186	14.97052674
LQ	3.5	0.904107746	1636.487155	12.43908191
LQ	4	0.902440141	1636.678698	12.63062549
H	0.5	0.981972535	NA	NA
H	1	0.967424648	NA	NA
H	1.5	0.958862676	NA	NA
H	2	0.94988662	NA	NA
H	2.5	0.939332394	NA	NA
H	3	0.93485	NA	NA
H	3.5	0.932688028	NA	NA
H	4	0.93028662	NA	NA
LQH	0.5	0.981790845	NA	NA
LQH	1	0.966676761	NA	NA
LQH	1.5	0.959626056	3007.031172	1382.983099
LQH	2	0.953407746	2037.420931	413.3728577
LQH	2.5	0.946280986	2056.566527	432.5184537
LQH	3	0.941428873	2827.640056	1203.591983
LQH	3.5	0.935476056	NA	NA
LQH	4	0.929333803	2051.988011	427.9399382
LQHP	0.5	0.985424648	NA	NA
LQHP	1	0.975890141	NA	NA
LQHP	1.5	0.968147183	NA	NA
LQHP	2	0.958862676	NA	NA
LQHP	2.5	0.952616197	NA	NA
LQHP	3	0.945390845	2498.261871	874.2137984
LQHP	3.5	0.93739507	2239.906526	615.8584527
LQHP	4	0.931835915	1756.168401	132.1203284

Table S5. Analysis of variable contributions for the current breeding habitats of the black-necked crane in Shaluli Mountains.

Variables	Percent contribution (%)	Permutaion importance
**bio_15**	**26.8**	**19.7**
**vegetation**	**21.1**	**0.9**
**slope**	**13.4**	**8.1**
**d_lake1**	**11.4**	**2.2**
**bio_2**	**8.8**	**1.3**
**bio_1**	**7.1**	**35.1**
d_residential	2.6	1.6
NPP	2.3	5.7
bio_17	2.2	11.1
bio_7	1.4	2.5
aspect	1.2	0.5
d_road	0.5	2.5
d_river1	0.5	3.6
elev	0.3	1.9
bio_8	0.3	2.6
bio_12	0.1	0.8

Table S6. Area of predicted distribution range for the black-necked crane at present and under future climate scenarios.

Scenario	Period	Total area (km^2)	Percent area changed	Gained area (km^2)	Lost area (km^2)	Stable area (km^2)	The area under Protected (km^2)	Percentage area under protected
Current		27122					6683	45.21%
SSP1-2.6	2021-2040	27200	0.29%	4655	4577	22545	6347	42.93%
	2041-2060	34870	28.57%	9355	1067	25515	8009	54.18%
	2061-2080	32852	21.13%	6971	1241	25881	7570	51.21%
	2081-2100	34763	28.17%	10032	2391	24731	7938	53.70%
SSP2-4.5	2021-2040	38401	41.59%	12064	785	26337	8487	57.41%
	2041-2060	30002	10.62%	6170	3290	23832	7111	48.10%
	2061-2080	40021	47.56%	13058	159	26963	8847	59.85%
	2081-2100	35359	30.37%	10426	2189	24933	8353	56.50%
SSP3-7.0	2021-2040	23912	-11.84%	2683	5893	21229	5887	39.82%
	2041-2060	30104	10.99%	7929	4947	22175	6644	44.94%
	2061-2080	30715	13.25%	7529	3936	23186	7451	50.40%
	2081-2100	30761	13.42%	9221	5582	21540	7395	50.20%
SSP5-8.5	2021-2040	32344	19.25%	8349	3127	23995	7341	49.66%
	2041-2060	34391	26.80%	9649	2380	24742	7941	53.72%
	2061-2080	35277	30.07%	10977	2822	24300	8243	55.76%
	2081-2100	35184	29.72%	11051	2989	24133	8417	56.94%

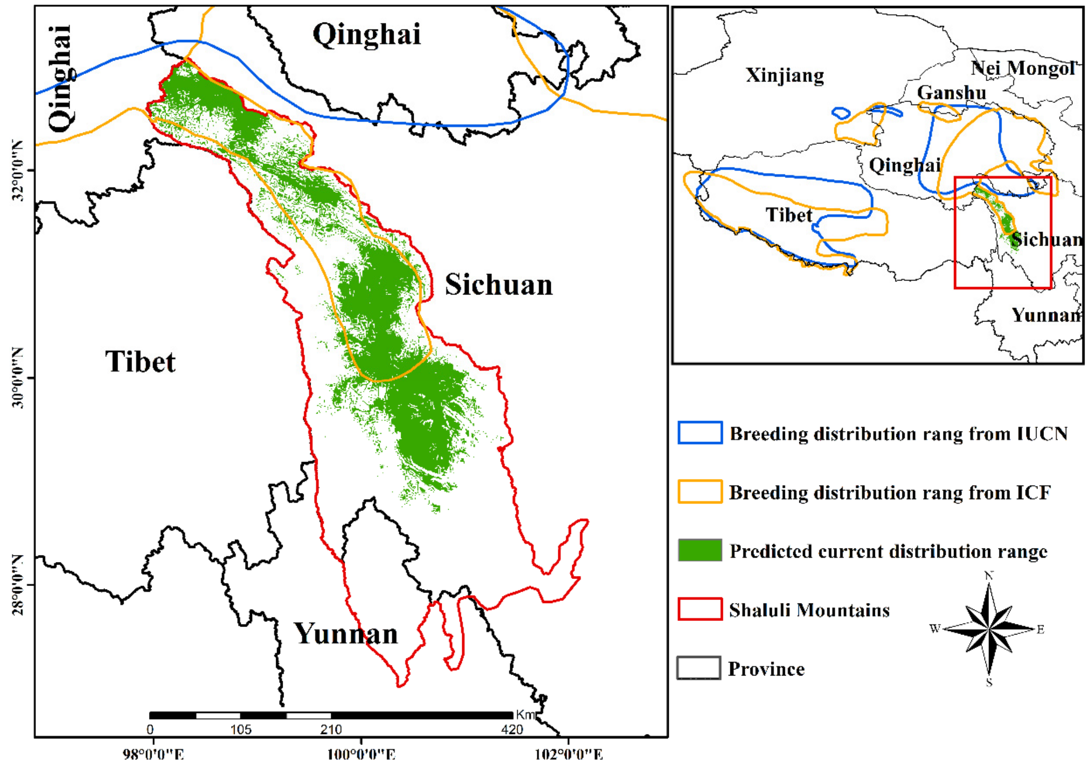

Figure S1. Current distribution range for the black-necked crane using MaxEnt, compared with the distribution range maps from IUCN (BirdLife International, 2020) and ICF (International Crane Foundation) (Li, 2019).

References

1. BirdLife International. 2020. Grus nigricollis. The IUCN Red List of Threatened Species 2020: e.T22692162A180030167 [Online]. [Accessed 15 April 2022].
2. LI, F. 2019. IUCN SSC Crane Specialist Group– Crane Conservation Strategy. In: MIRANDE CM & HARRIS JT (eds.) SPECIES REVIEW:BLACK-NECKED CRANE (*Grus nigricollis*). International Crane Foundation Baraboo, Wisconsin, USA.

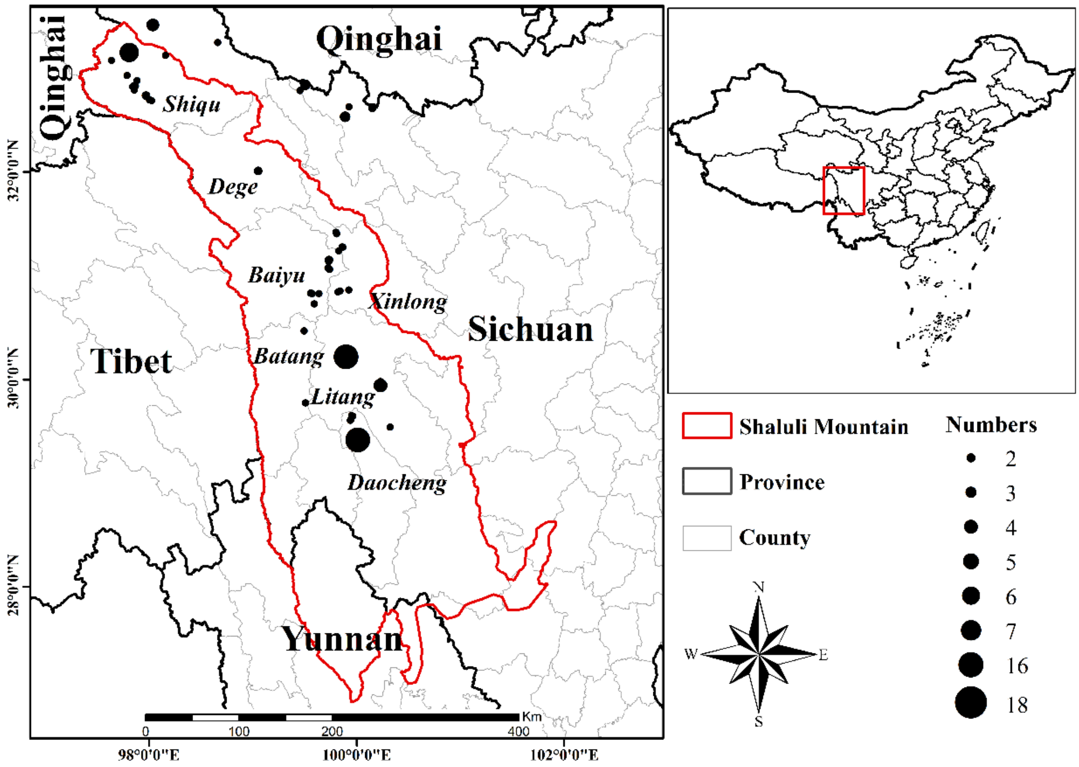

Figure S2. Distribution of breeding black-necked crane survey sites in the Shaluli Mountains. Black points indicate the maximum number recorded in a single survey in that area.
